# Are comorbidities associated with the cytokine/chemokine profile of persistent apical periodontitis?

**DOI:** 10.1007/s00784-023-05139-3

**Published:** 2023-07-11

**Authors:** Anne Eriksson Agger, Janne Elin Reseland, Erik Hjelkrem, Aina-Mari Lian, Else K. Breivik Hals, Homan Zandi, Pia Titterud Sunde

**Affiliations:** 1 Department of Biomaterials, Institute of Clinical Dentistry, University of Oslo, Oslo, Norway; 2 Department of Endodontics, Institute of Clinical Dentistry, University of Oslo, Oslo, Norway; 3 TAKO-Centre, National Resource Centre for Oral Health in Rare Medical Conditions, Lovisenberg Diakonale Hospital, Oslo, Norway; 4 Department of Endodontics, Institute of Clinical Dentistry, University of Oslo, Postboks 1109 Blindern, N-0317 Oslo, Norway

**Keywords:** Persistent Apical Periodontitis, Arthritis, Cytokines, Immunology, Periodontal Disease

## Abstract

**Objectives:**

This study aimed to identify disease-related markers in persistent apical periodontitis (PAP) biopsies and examine whether these were associated with comorbidities like rheumatoid arthritis (RA) and cardiovascular diseases (CVD).

**Materials and method:**

The levels of the cytokines/chemokines GM-CSF, IFN-γ, IL-2, IL-6, IL-9, IL-10, IL-13, IL-15, IL-17E/IL-25, IL-21, IL-23, IL-27, IL-28A/IFN -λ2, IL-33, MIP-3α/CCL20, and TNF-α were determined in lesions from patients with PAP (*n* = 20) and compared to healthy bone samples (*n* = 20).

**Results:**

We identified eleven cytokines to be differently expressed, and among them, IL-2, IL-6, IL-17E, IL-21, and IL-27 appeared to drive the discrepancy between the disease and healthy groups. The levels of T follicular helper (Tfh) cell promoting cytokines (IL-21, IL-6, IL-27) were enhanced while T helper (Th) 1 cell promoting cytokine (IL-2), Th2 cell promoting cytokine (IL-13), and Th17 cell promoting cytokine (IL-17E) were reduced in the PAP group. The data also indicate that Tfh cell differentiation (IL-21), along with Th1 (GM-CSF, IFNγ), Th2 (IL-13), and Th17 (GM-CSF) cell differentiation, might be increased in the subpopulation of patients suffering from RA, whereas no differences were found in patients with CVD.

**Conclusions:**

Levels of cytokines/chemokines in PAP were identified, and cluster analyzes indicated that these markers may be associated with the differentiation of different T cell populations. Patients with PAP and RA comorbidities showed elevated levels of markers reinforcing this association.

**Clinical relevance:**

Molecular analyses of PAP may result in identification of prognostic markers.

## Introduction

Root canal infection results in breakdown of the periapical bone and formation of an apical lesion which is manifested on X-ray by the formation of a periapical radiolucency. An apical lesion is a mass of inflamed granulation tissue surrounded by an outer capsule of dense fibrous tissue [1]. Granulation tissue contains a variety of cells including immune cells such as T cells, B cells, and macrophages.

At dental educational institutions and in specialist practice, a success rate of endodontic treatment of 75–85% is shown in teeth with preoperative apical periodontitis (AP) [2–6]. Persistent apical periodontitis (PAP) is a chronic inflammatory disease that occurs when root canal treatment has not adequately eliminated the infection. PAP can be asymptomatic or symptomatic and is most often diagnosed by observing visible lesions on X-ray. Teeth with PAP can be treated by either nonsurgical endodontic retreatment or periapical surgery. Large apical lesions (> 5 mm) have showed lowered healing rate compared to smaller lesions (< 5 mm) [5, 7] and teeth with lesions > 5 mm are more likely to be extracted [8]. Large lesions are also correlated with development of cysts [9].

The resorption of periapical bone, caused by an imbalance between osteoblast and osteoclast activity, may be modulated by cytokines [10]. Cytokines are signaling molecules that most cells produce in response to changes in their environment [11]. Cytokines have multifaceted roles [12]. Interleukin (IL)-6 may be involved in the pathogenesis of apical lesions, chronic inflammation, differentiation of naïve T cells to specific T helper cells [13], and bone remodeling [14]. However, the role of IL-6 in bone turnover is twofold, as IL-6 acts as an osteoclast modulator [15] and promotes osteogenic differentiation [16].

The pathogenesis of PAP is characterized by an inflammatory infiltrate of immune cells, mostly T cells, B cells, and macrophages [17]. During inflammation, naïve T cells differentiate into CD4^+^ T helper (Th) cells: Th1, Th2, Th9, Th17, Th22, regulatory T (Treg) cells, and T follicular helper (Tfh) cells [18]. These cells are stimulated by cytokines from the immune response and, in turn, regulate the immune system through cytokine release [19]. The cytokine networks involved in the pathogenesis of PAP can be identified by simultaneous analysis of a wide range of cytokines. Inflammatory comorbidities may affect which cytokines are present and the levels of these in PAP, but it is unclear whether different comorbidities, like rheumatoid arthritis (RA) and cardiovascular disease (CVD) affect the cytokine profiles differently.

Rheumatoid arthritis (RA) and cardiovascular diseases (CVD) are both inflammatory diseases. RA is an autoimmune and inflammatory disease that causes the immune system to attack normal cells in the body. It leads to inflammation and swelling of the joints with continued expression of inflammatory cytokines like IL-6 and IL-21 [20]. Previous studies have shown an association between arthritis and apical periodontitis [21–23] as patients suffering from arthritis are more likely to develop apical periodontitis and thus at higher risk for PAP.

Cardiovascular disease (CVD) is a general term for disorders of the heart and blood vessels and is characterized by increased expression of IL-6 and tumor necrosis factor-α (TNFα) [24]. Multiple studies and reviews have shown an association between CVD and apical periodontitis [25–27]. A systematic review found that CVD may be correlated with endodontic outcome [28].

The aim of the present study was to identify cellular markers and evaluate the levels of inflammatory cytokines in PAP. Furthermore, to investigate if comorbidities affected the expression levels of cytokines. The association between lesion size and cytokine expression as well as the presence of pre-operative symptoms, affected teeth, and demographic information of the patients were also investigated.

## Method

### Patient characteristics and sample collection

Twenty patients (9 women and 11 men) with an average age of 53.5 years (± 14.9) with PAP were referred to an experienced endodontist in private practice for the treatment of one tooth each. The patients were without sinus tracts or endo-perio-like lesions, and none had previously responded to conventional endodontic therapy. Information about medications was collected prior to treatment from each patient, and their anti-inflammatory effects were evaluated based on literature available. Among these, were metformin, reported for the two diabetes patients; atorvastatin, reported by one CVD patient; and diclofenac, reported by one arthritis patient.

Teeth with vertical root fractures were excluded. Clinical inspection showed that all teeth had satisfactory coronal restorations. Radiographically, it was evident that the patients referred for treatment had a root-filled tooth with periapical radiolucencies of diameters varying between 5.0 and 14.5 mm. Lesions were divided into two groups: 5–10 mm (*n* = 12) and > 10 mm (*n* = 8) [29]. Ten patients had symptoms such as tenderness to percussion and biting at the time of surgery. Teeth treated were molars (*n* = 5), premolars (*n* = 6), canine (*n* = 1), and incisors (*n* = 8). Ten patients had no underlying diseases, while three suffered from RA, four suffered from CVD, two suffered from diabetes, and two suffered from psychological disorders. One patient suffered from both CVD and psychological disorders and is included in both groups. Demographic and clinical data are listed in Table 1.

**Table 1:** Clinical and demographic data of the PAP patients

Patients	PAP
n	20
Age, mean ($\pm$ SD)	53.5 ($\pm$ 14.9)
Gender, Female / Male	9 / 11
Pre-operative, symptoms yes / no	10 / 10
Lesion size range, mm	5.0 – 14.5
Lesion size, n:	
5–10 mm	12
> 10 mm	8
Teeth, n:	
Incisor	8
Canine	1
Premolar	6
Molar	5
Comorbidity, n:	
None	10
Cardiovascular Disease (CVD)	4*
Rheumatoid Arthritis (RA)	3
Diabetes	2
Psychological disorders	2

Data presented as mean ± standard deviation. *) one patient in this group also suffered from psychological disorders and is counted in both groups

All patients had an oral rinse with 0.2% chlorhexidine gluconate solution for 30 s immediately before surgery. A full-thickness muco-periosteal flap was reflected and the whole lesion was enucleated before apicectomy. Care was taken to avoid contamination from saliva.

Healthy crestal and trabecular bone tissue collected from healthy patients during surgical removal of the third molars due to horizontal impaction were used as controls (*n* = 20). All teeth had to be healthy for the samples to be included in the control group. An experienced oral surgeon in private practice collected all control samples.

All samples were washed in PBS to remove blood and dried on sterile surgical gauze. They were snap-frozen in liquid nitrogen, before storage at -80 °C prior to analyses.

### Protein extraction and quantification

Samples from both PAP patients and controls were kept on ice throughout protein extraction. Each sample was weighed, and pulverized in a pre-chilled cryocrusher (Spectrum™ Bessman Tissue Pulverizers 10–50 mg capacity) kept in liquid nitrogen. Trizol (Thermo Fisher Scientific, Waltham, MA, USA) was added in a ratio of 1 mL per 100 mg pulverized tissue, and proteins isolated according to the manufacturer’s protocol. In brief, the organic and aqueous phases were separated with chloroform (25,669; Fluka, Buchs, Switzerland). Proteins were precipitated with isopropanol. Precipitates were washed with 0.3 M Guanidine HCL (G3272; Sigma Aldrich, Poole, UK) in 95% ethanol (20,821.310; VWR Chemicals, Radnor, PA, USA). All samples were air-dried and stored at -80 °C.

The proteins were resuspended in 1% SDS at 50 °C, and further diluted to final concentration of 0.1% SDS. Total protein was quantified using Micro BCA (Pierce™ BCA Protein Assay Kit, Sigma Aldrich) following the manufacturer’s instructions, using serial dilutions of bovine serum albumin as standards. The total protein content was calculated in % of 50 mg extracted, pulverized tissues.

The protein samples were analyzed for 25 cytokines using Milliplex Multi-Analyte Profiling Human TH17 Panel (Merck, Kenilworth, NJ, USA). Granulocyte–macrophage colony-stimulating factor (GM-CSF), interferon gamma (IFN-γ), interleukin 1 beta (IL-1β), interleukin 2 (IL-2), interleukin 4 (IL-4), interleukin 5 (IL-5), interleukin 6 (IL-6), interleukin 9 (IL-9), interleukin 10 (IL-10), interleukin 12p70 (IL-12(p70)), interleukin 13 (IL-13), interleukin 15 (IL-15), interleukin 17A (IL-17A), interleukin 17F (IL-17F), interleukin 17E (IL-17E/IL-25), interleukin 21 (IL-21), interleukin 22 (IL-22), interleukin 23 (IL-23), interleukin 27 (IL-27), interleukin 28A (IL-28A/IFN-λ2), interleukin 31 (IL-31), interleukin 33 (IL-33), macrophage inflammatory protein 3 alpha (MIP-3α/CCL20), tumor necrosis factor alpha (TNF-α), and tumor necrosis factor beta (TNFβ) were analyzed using HTH17MAG-14 K (Merck). The analytes were quantified from the mean fluorescent intensities using the Luminex 200 system (Luminex, Austin, TX, USA) running Luminex xPONENT 3.1 software. Concentrations were calculated using a logistic 5P weighted standard curve based on reference analyte gradient concentrations. To standardize the concentrations, they were divided by the total protein concentration.

### Statistical analyzes and visualization

All statistical analysis was performed using SigmaPlot version 14 (Systat Software, San Jose, CA, USA). The Shapiro–Wilk test was used to examine whether the data were normally distributed. In cases where normality and equality were met, Student’s t-test was performed. In cases where normality or equality were not met, Mann–Whitney Rank Sum test was performed. Differences were considered significant with a probability of $\le$ 0.05.

When investigating the association of comorbidity, only diseases with a minimum of three patients were used. There were only two patients with diabetes and psychological disorders, respectively, consequently those were not investigated.

Plots and graphs were made in R (version 4.1.2) within the RStudio platform (version 2021.9.0.351). Violin plots were made running the ggplot2 package [30].

Principal component analysis (PCA) was performed using ggfortify [31]. PCA is an unsupervised method for data reduction that allows identification and ranking of the major sources of variation in a multi-dimensional dataset without introducing inherent bias. Principal components are new variables that are constructed as linear functions of the initial variables and which explain the maximum amount of variance in the data. These principal components are uncorrelated and the information is compressed so that the least amount of principal components explain the most amount of the original data. Here, PCA was used to evaluate the relevance of each of the statistically different cytokines in separating the healthy from the diseased subjects and separating the diseased group based on comorbidities. Using the Kaiser criterion, only those principal components with an eigenvalue over 1.0 were included.

Cluster heatmaps were made running gplots [32] and RColorBrewer [33]. Cluster heatmaps is an unsupervised method for identifying patterns of clustering in a multi-dimensional dataset. Here, cluster heatmaps were used to investigate expression of all the measured analytes and the pattern of clustering among them on the basis of all samples.

## Results

### Higher total protein concentration in PAP lesions

**Fig. 1:**
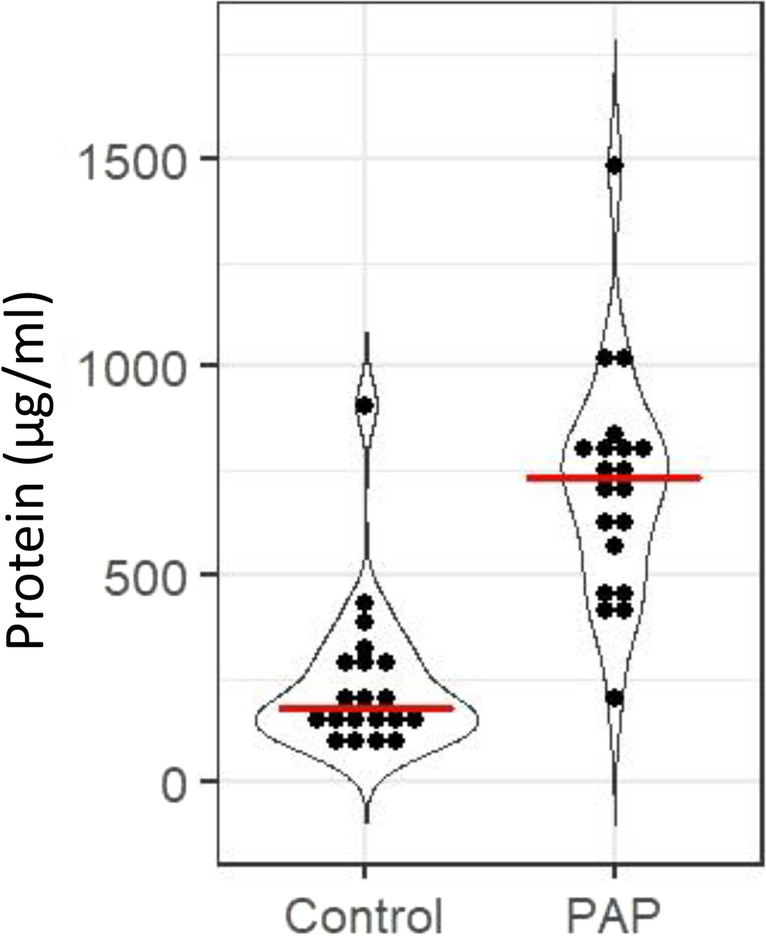
Total protein concentration in healthy controls and diseased PAP patients. Dots represents each subject in each group. Healthy control, *n* = 20; PAP, *n* = 20. Red lines shows the median. Data presented as protein in µg/ml

The total protein concentration was significantly elevated in PAP lesions compared to healthy bone (*p* $\le$ 0.001) (Fig. 1). There was a significant (*p* $\le$ 0.001) difference in total protein content in PAP lesions (73 $\pm$ 28%) versus healthy bone (24 $\pm$ 19%).

There were no differences in the total protein concentration based on lesion size. There was also no differences in protein concentration in patients with no underlying diseases vs patients with RA or CVD.

### Elevated levels of putative bone modulating cytokines in PAP lesions

**Fig. 2:**
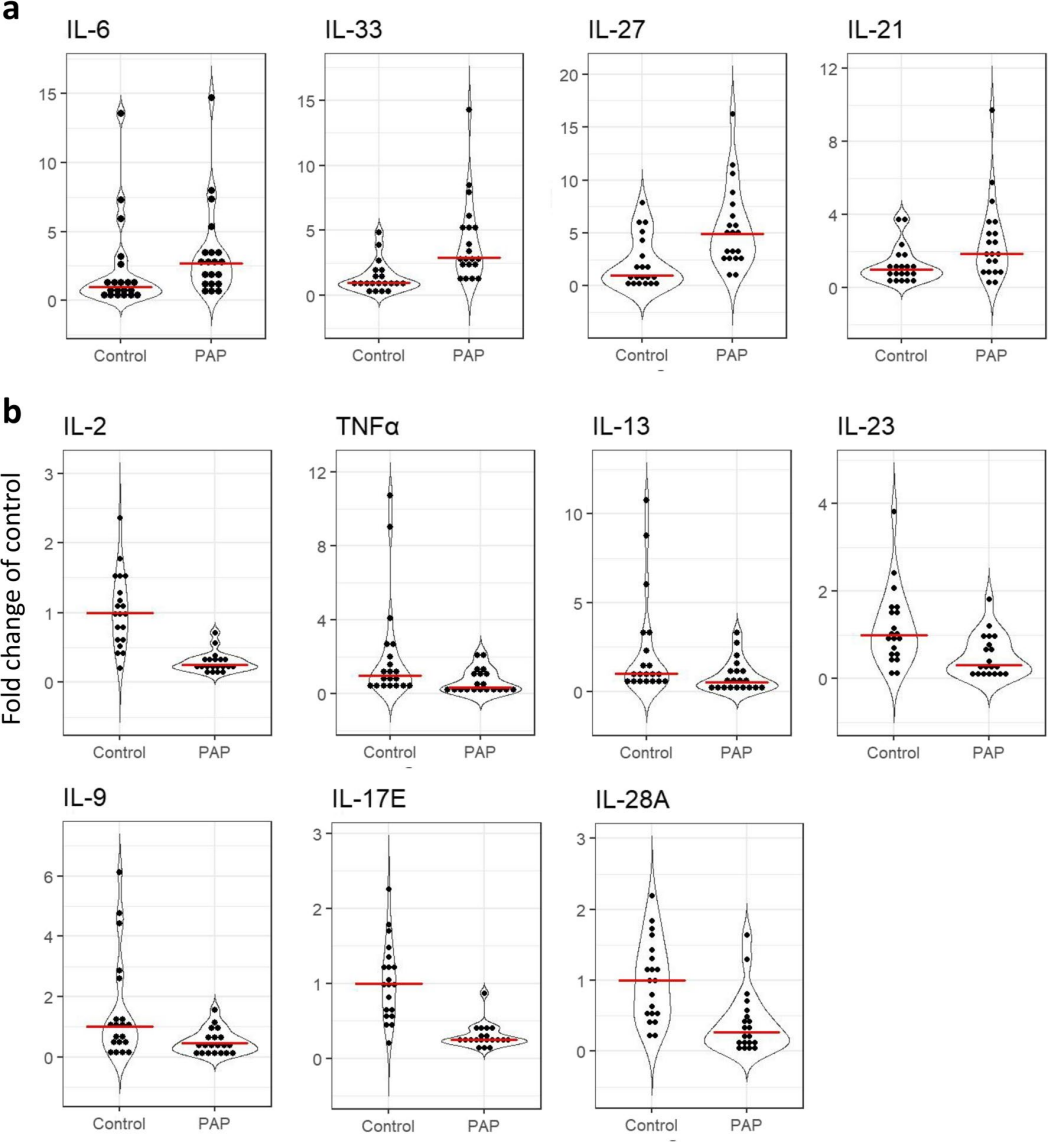
Cytokine detection in healthy bone and PAP lesions. **a:** Cytokines with putative osteoblast promoting activity. **b:** Cytokines involved in other bone remodeling activity. Dots represents each subject in either the healthy (*n* = 20) or the diseased PAP (*n* = 20) group. The red lines shows the median. Only statistically different cytokines with a probability of < 0.05 are shown. Data presented as fold change of control

The levels of IL-6 (*p* = 0.015), IL-21 (*p* = 0.022), IL-27 (*p* = 0.001), and IL-33 (*p* $\le$ 0.001), were increased in PAP lesions compared to healthy bone (Fig. 2a). Whereas the levels of IL-2 (*p* $\le$ 0.001), IL-9 (*p* = 0.026), IL-13 (*p* = 0.019), IL-17E (*p* $\le$ 0.001), IL-23 (*p* = 0.004), IL-28A (*p* $\le$ 0.001), and TNFα (*p* = 0.006) were significantly reduced in PAP lesions compared to healthy bone (Fig. 2b).

The levels of GM-CSF, IFNγ, IL-10, CCL20, and IL-15 were found not to be significantly different between the healthy bone and PAP lesions. The levels of IL-1β, IL-4, IL-5, IL-12(p70), IL-17A, IL-17F, IL-22, IL-31, and TNFβ were below the detection limit for the kit, and thus not included in the analysis.

### Principal component analysis separate PAP samples from control samples

**Fig. 3:**
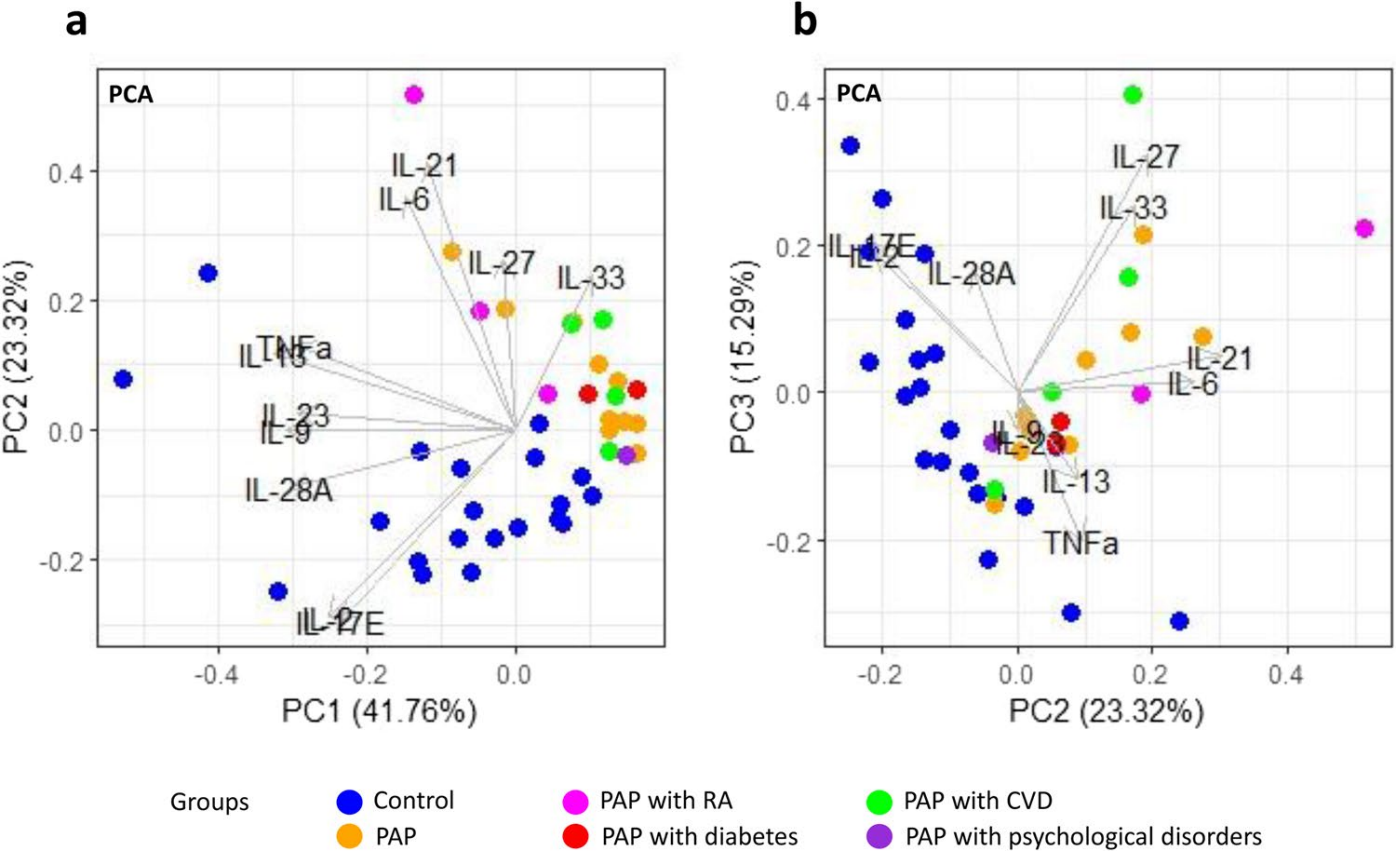
Principal components analysis (PCA) depicting healthy bone and PAP lesions with both scores and loadings. PC1 vs PC2 (**a**) and PC2 vs PV3 (**b**). Color key indicates the groups: blue: healthy bone (*n* = 20), orange: PAP with no underlying diseases (*n* = 10), pink: PAP + RA (*n* = 3), red: PAP with diabetes (*n* = 2), green: PAP with CVD (*n* = 4), and purple: PAP with psychological disorders (*n* = 2) one patient in this group also suffered from CVD and is only visualized as CVD. Only significantly different cytokines were used for the PCA

Principal component analysis was performed to identify the principal components that accounted for the majority of the variation of the levels of the cytokines. Principal component (PC) 1 explained 41.76% of the variation in the data, while PC2 explained 23.32% and PC3 explained 15.29%, bringing the total explained variation to 80.36%. Figure 3a shows PC1 vs PC2 and Fig. 3b PC2 vs PC3. There was a clear separation of healthy bone (blue) and PAP lesions (orange: PAP with no comorbidity, pink: PAP with RA, red: PAP with diabetes, green: PAP with CVD, and purple: PAP with psychological disorders) along PC2, slightly along PC1, but not along PC3. There was no separation within the PAP group based on comorbidity. Cytokines with a loadings score in PC2 $\le$ −0.5 or $\ge$ 0.5 were considered responsible for the separation and those were identified as IL-2, IL-6, IL-17E, IL-21, and IL-27. Among these variables, there was strong covariation between IL-21 and IL-6 as well as between IL-2 and IL-17E.

### Pattern of cytokine clustering is different in PAP lesions compared to healthy bone

**Fig. 4:**
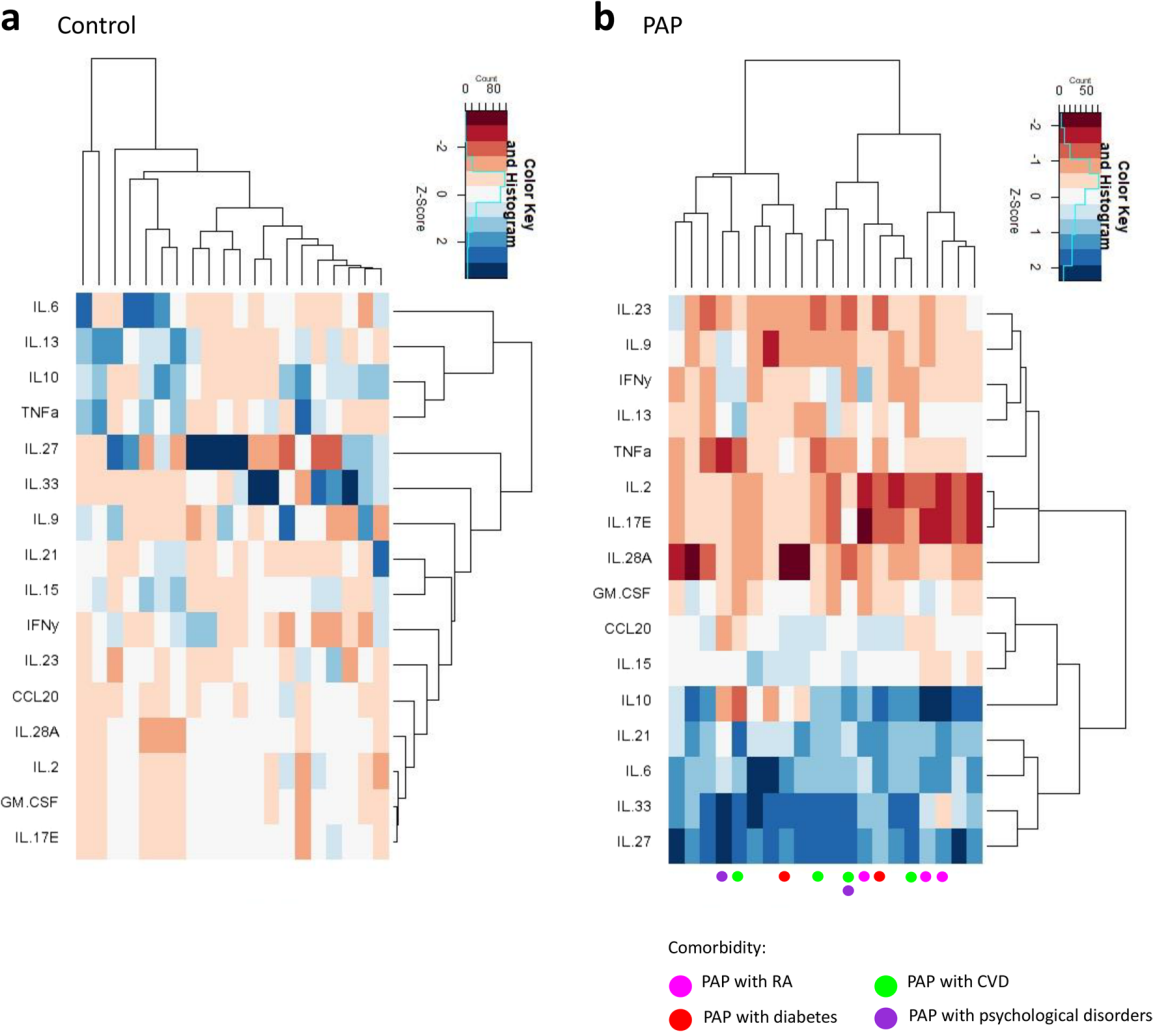
Heatmaps over cytokine expression in healthy bone and PAP lesions. Data presented as log2 fold change of the median of the control samples and scaled to subjects. Rows: cytokines, columns: individual samples from control (**a**) (*n* = 20) and PAP (**b**) (*n* = 20). Color key indicates expression values autoscaled to z-scores: dark red: lowest, dark blue: highest. Dots indicate the subjects with comorbidity: pink: PAP + RA (*n* = 3), red: PAP + diabetes (*n* = 2), green: PAP + CVD (*n* = 4), and purple: PAP + psychological disorders (*n* = 2)

Clustered heatmaps of all measurable cytokines in each sample are presented in Fig. 4. In both the healthy bone (Fig. 4a) and the PAP lesions (Fig. 4b), the cytokines clustered into two clusters. However, the composition of the clusters were different. In healthy bone, the first cluster contained IL-6, IL-13, IL-10, and TNFα, while the second cluster contained IL-27, IL-23, IL-9, IL-21, IL-15, IFNγ, IL-23, CCL20, IL-28A, IL-2, GM-CSF, and IL-17E. In the PAP lesions, the two clusters are of equal size with IL-23, IL-9, IFNγ, IL-13, TNFα, IL-2, IL-17E, and IL-28A in the first cluster and GM-CSF, CCL20, IL-15, IL-10, IL-21, IL-6, IL-33, IL-27 in the second cluster. When investigating comorbidity there was no clustering in the heatmap (dots in Fig. 4b).

### PAP lesions in patients with RA, have a different cytokine pattern than PAP lesions from ‘healthy’ patients

**Fig. 5:**
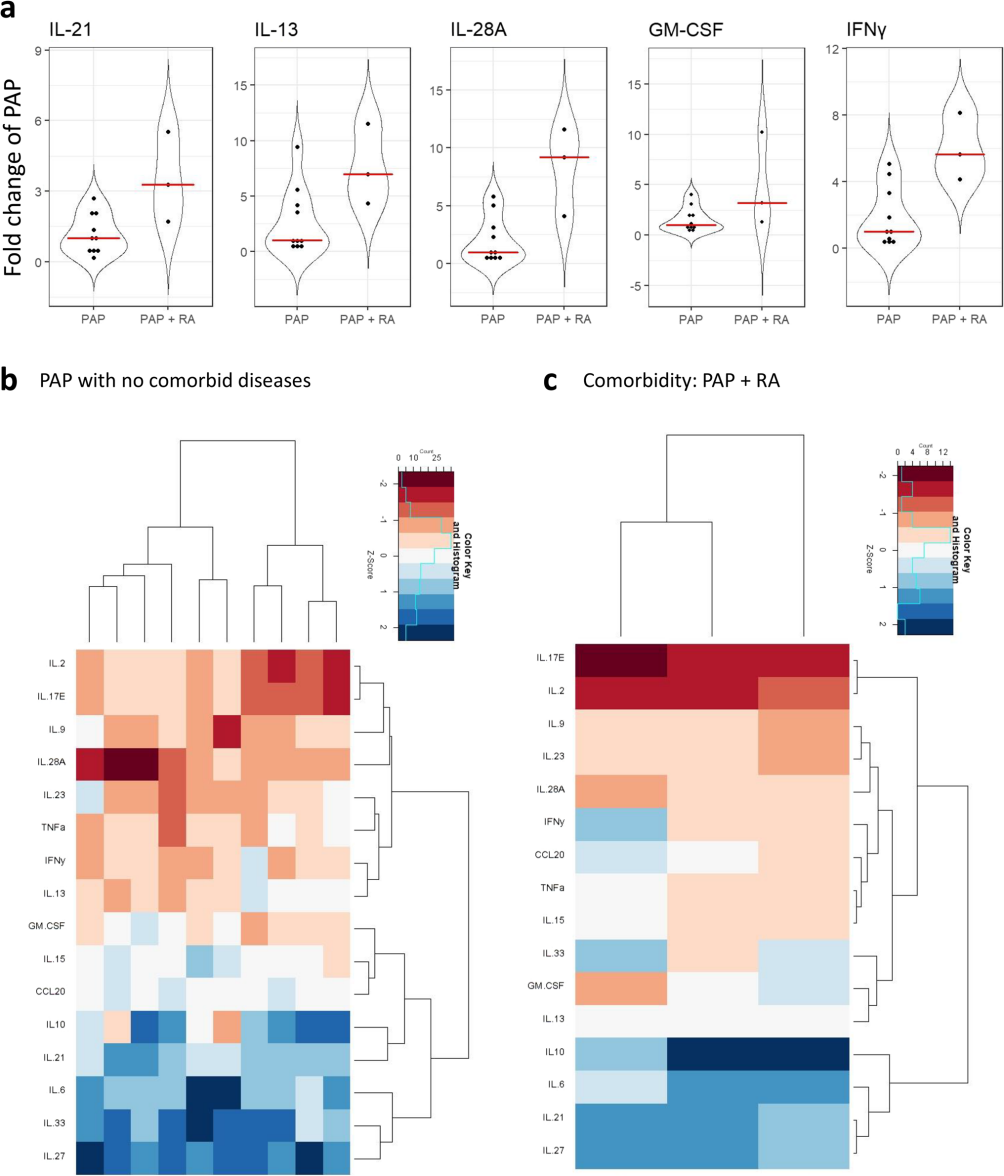
Cytokine expression in PAP lesions from patients without comorbidities and PAP lesions from patients with RA comorbidity. **a**: Cytokine detection. Dots represents each subject in either PAP lesions (*n* = 10) or PAP lesions from patients with RA comorbidity (*n* = 3). The red line shows the median. Only statistically different cytokines with a probability of $\le$ 0.05 are shown. Data presented as fold change. Heatmaps for PAP patients (**b**) and PAP patients with RA comorbidity (**c**). Data presented as log2 fold change of the median of the control samples and scaled to patients. Rows: cytokines, columns: individual samples. Color key indicates expression values autoscaled to z-scores: dark red: lowest, dark blue: highest

To better understand if comorbidity affected the cytokine levels, PAP with RA and PAP with CVD were compared to PAP lesions in healthy patients. Patients with RA comorbidity had significant differences in cytokine levels of IFNγ (*p* = 0.035), GM-CSF (*p* = 0.047), IL-13 (*p* = 0.036), IL-21 (*p* = 0.009), and IL-28A (*p* = 0.002) (Fig. 5a) compared to PAP lesions from patients with no underlying diseases. Compared to control samples on the other hand, PAP with RA had a significantly enhanced level of only IL-21 (*p* = 0.012).

There were no differences in cytokine expression in PAP with CVD compared to PAP with no comorbidities.

In PAP with no comorbidity and PAP with RA, the cytokines appeared to cluster differently. In PAP with no comorbidity, the first cluster contained IL-2, IL-17E, IL-9, IL-28A, IL-23, TNFα, IFNγ, IL-13, while the second cluster contained GM-CSF, IL-15, CCL20, IL-10, IL-21, IL-6, IL-33, and IL-27 (Fig. 5b). For PAP with RA the first cluster contains IL-17E, IL-2, IL-9, IL-23, IL-28A, IFNγ, CCL20, TNFα, IL-15, IL-33, GM-CSF, IL-13, while the other cluster contained IL-10, IL-6, IL-21, and IL-27 (Fig. 5c).

### Lesion size varied related to preoperative symptoms, tooth type, and comorbidity, however cytokine levels were not affected

There were no statistical differences in the cytokine expression related to symptoms, lesion size, tooth type, age, nor gender.

**Fig. 6:**
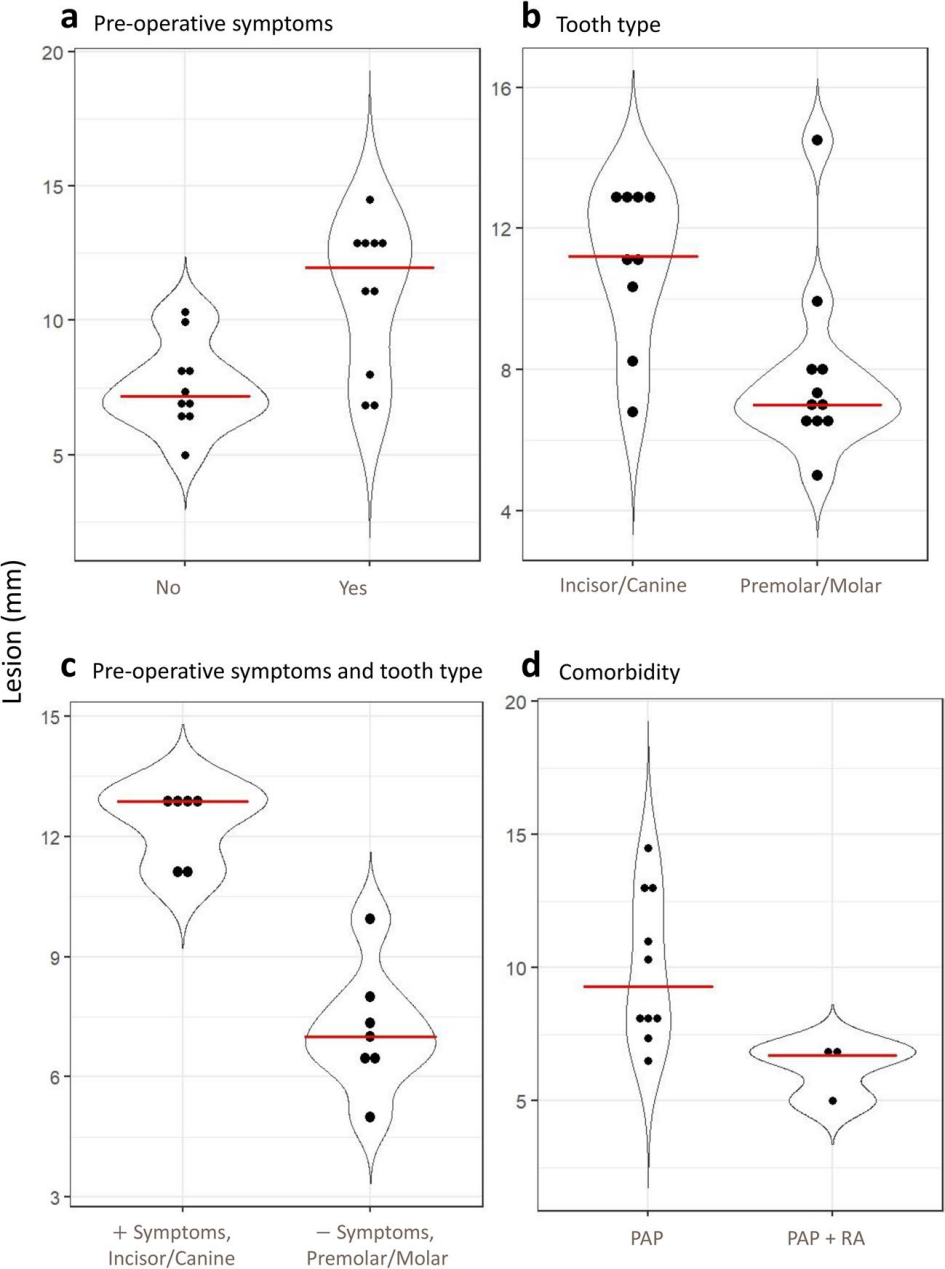
Clinical information in relation to lesion size. **a:** Symptoms pre-operation: No (*n* = 10), Yes (*n* = 10). **b**: Tooth type: Incisor + Canine (*n* = 9), Premolar + Molar (*n* = 11). **c**: Combination of symptoms and tooth type: With symptoms in incisor and canine teeth (*n* = 6), without symptoms in premolar and molar teeth (*n* = 7). **d**: Lesion size in relation to comorbidity: PAP (*n* = 10), PAP + RA (*n* = 3). Dots represents each subject. Red lines shows the median. Data presented as lesion size in mm. Only statistically different characteristics with a probability of < 0.05 are shown

Preoperative symptoms and tooth type were able to separate samples on lesion size. Patients with preoperative symptoms (*p* = 0.003) as well as incisors and canines (*p* = 0.009) had larger lesions. The asymptomatic group had smaller lesions (7.2 $\pm$ 1.2 mm) than the symptomatic group (12.4 $\pm$ 1.4 mm) (Fig. 6a). Incisors and canines had larger lesions (11.04 $\pm$ 2.3 mm) and premolars and molars had smaller lesions (7.9 mm $\pm$ 2.5 mm) (Fig. 6b).

When comparing both tooth type and pre-operative symptoms with lesions size, only incisors and canines with symptoms and premolars and molars without symptoms were significantly different (*p* = 0.008). Incisors and canines with symptoms had larger lesions (12.3 $\pm$ 0.8 mm) and premolars and molars without symptoms had smaller lesions (7.2 mm $\pm$ 0.8 mm) (Fig. 6c). Incisors and canines without symptoms had moderate lesions (8.5 $\pm$ 1.2 mm) as had premolars and molars with symptoms (9.1 $\pm$ 1.0 mm).

RA had effect on lesion size (*p* = 0.048) with patients with no comorbidity having larger lesions (9.9 $\pm$ 2.8 mm) and patients with PAP and RA comorbidity having smaller lesions (6.2 $\pm$ 1.1 mm) (Fig. 6d), while CVD had no effect on lesion size.

## Discussion

In the present study, cytokine profiles were differentiated between patients with PAP lesions and healthy bone tissue from healthy controls. However, cytokines could not be used to differentiate between patients with PAP lesions, with or without symptoms nor between the sizes of the lesions.

Previous studies have shown increased IL-6, IL-21, IL-27, and IL-33 in PAP lesions [34–36], which is in accordance with the results presented here. These cytokines have also been shown to have osteogenic activity [14, 37–39]. Among the seven cytokines that we found reduced in PAP lesions, IL-9, IL-23, and TNFα have previously been found to be increased in PAP lesions [34]. The roles of IL-2, IL-13, IL-17E, and IL-28A in PAP lesions are less understood, however, increased IL-2 [40], IL-13 [41], and IL-28A [42] and decreased IL-17E [43] levels are associated with marginal periodontitis. All cytokines identified to be reduced in this study, IL-2, IL-9, IL-13, IL-17E, IL-23, IL-28A, and TNFα, have been associated with osteoclast promoting activity [44–48]. This indicates increased signaling for bone formation and decreased bone resorption. Apical inflammation is associated with bone loss [49]. Xu and colleagues [50], performed histological staining of PAP lesions and found decreased bone tissue and instead increased soft tissue characteristics like blood vessels and collagen fibers. The protein content in soft tissue is considerably higher than the protein content in bone [51], and the calculated levels in our PAP samples correspond to the protein content found in soft and granulation tissue. Indicating a degradation of mineralized tissue and an invasion of cells [52–54]. Based on the PCA plots, the cytokines that explain the most amount of the variation between healthy and diseased individuals were identified, while cluster heatmaps showed the cytokines cluster differently in healthy bone versus PAP lesions. Not only are the tissue types different between healthy bone and PAP lesions, but the roles of the cytokines are no longer activating bone turnover, meaning the cytokines undergo a role change in PAP lesions compared to healthy bone.

In cell signaling, cytokines have multifaceted roles [55]. IL-6 is a pro-inflammatory cytokine involved in chronic inflammation [12] along with TNFα [56]. Yet, here there was increased IL-6 but decreased TNFα, which indicates the signaling is not purely inflammatory. This study showed strong covariation between IL-6 and IL-21 in diseased individuals, which indicates the cytokines are linked in the pathogenesis of PAP. IL-6 is essential for the differentiation from CD4^+^ T cells to specific effector T cell subsets—in particularly to T follicular helper (Tfh) cells while IL-21 is a product of Tfh cells [57]. IL-27, which cluster with IL-6 and IL-21, enhances IL-21 production by Tfh cells [58] while also recognized to reduce Th1 [59] and Th2 [60] cell response as well as inhibiting IL-17 production by Th17 cells. IL-2 (marker for Th1), IL-13 (marker for Th2), IL-17E (marker for Th17) were decreased in this study. IL-33, on the other hand, is involved in B cell activation [61]. Mature Tfh cells migrate to B cell follicles and provide essential signaling for B cell proliferation, isotype switching, and somatic hypermutation to plasma cells and memory B cells [62]. Taken together, our results suggest the pathogenesis of PAP is regulated by increased Tfh cell differentiation and B cell activation and decreased Th1, Th2 and Th17 cell differentiation.

RA is a chronic autoimmune inflammatory disease that is characterized by synovitis and bone erosions, which is driven by wide variety of immune cells. Among them, T cells are essential as disturbances in the balance of T helper cells play a crucial part of the pathogenesis of RA [63]. PAP lesions from patients with RA comorbidity had higher expression of IL-21 compared to PAP lesions from patients with no comorbidity and healthy bone samples. This suggests that Tfh cells are also crucial for the pathogenesis of PAP and RA comorbidity. IL-21 is not the only cytokine found increased in the patients with comorbidity. In addition, IL-13, IL-28A, GM-CSF, and IFNγ were increased in the comorbid patients. IL-13, an anti-inflammatory cytokine produced by both natural killer cells and T cells, is a B cell stimulating factor [64], and recognized as crucial for the pathogenesis of autoimmune rheumatic diseases, including RA [65, 66]. IL-13 is mainly produced by Th2 cells. GM-CSF is an inflammatory cytokine with roles as growth and differentiation factor for granulocytes and macrophages. A wide range of cells, both hematopoietic and non-hematopoietic, produce GM-CSF—Th1 and Th17 cells in particular [67, 68]. Elevated levels of GM-CSF are associated with RA [69]. Interferons are crucial for host defense, but can also contribute to the pathogenesis of inflammatory diseases. IFNγ, a type II interferon, is mainly produced by Th1 cells [70] and increased levels of IFNγ is associated with RA [71]. IL-28A also known as IFN-λ2, is a type III interferon and one of the newer players recognized in the pathogenesis of RA [72] with important roles in regulating neutrophils [73]. IL-28A is produced by dendritic cells and macrophages [74]. These results suggest that arthritis might affect the pathogenesis of PAP by not only increased Tfh cell differentiation but also Th1, Th2, and Th17 cell differentiation. However, this would need to be investigated further due to the limited number of patients suffering from PAP and RA comorbidity. Patients with PAP and other inflammatory diseases such as diabetes type II may also be of interest to investigate.

The cytokine profiles from PAP lesions were compared to healthy crestal and trabecular bone tissues from patients prone to tooth extraction. Identifying and enrolling patients with RA needing tooth extraction was difficult. Due to ethical considerations, bone tissue from patients without PAP but suffering from diseases like RA without need for tooth extraction could not be included.

Due to the small size of PAP lesions from each individual, there was only enough material to perform protein analysis.

Patients may have taken medication that affects their cytokine/chemokine profile prior to sample harvesting [75]. We cannot exclude an effect of medication on our results, but there were no clear trend in the type or distribution of medication between PAP and PAP with comorbidity patients, except for the diabetes group. Diabetes is not included as comorbidity due to the low number of patients.

**Fig. 7:**
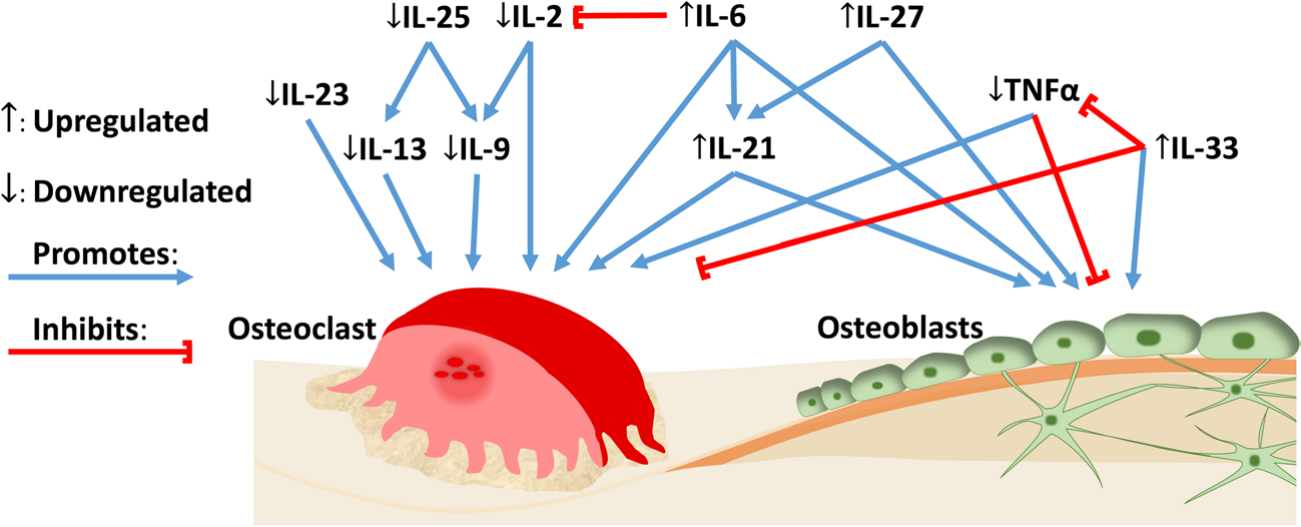
Overview of cytokine activity on osteoclasts and osteoblasts with regulation as found in this study

We identified different levels of cytokines in PAP samples compared to controls (Fig. 7). To determine a prognostic marker or markers, a consecutive follow-up of patients after apical surgery are needed.

## Conclusion

In conclusion, this study identified eleven cytokine/chemokine markers in PAP and a cluster of markers that might be associated with the differentiation of various T cell populations. Patients with PAP and RA comorbidities had elevated levels of five markers compared to PAP patients without comorbidities. Further, there were no association between cytokine profiles and the size of apical lesions, the presence of pre-operative symptoms or the affected teeth.

## Data Availability

Data supporting the findings of this study can be made available upon request from the corresponding author, PTS. These data will not be made publicly available due to ethical considerations, as information that could compromise the privacy of enrolled patients are included.
